# Visualizing epidemiological models for policy: design principles for effective communication

**DOI:** 10.3389/fpubh.2026.1798154

**Published:** 2026-05-14

**Authors:** Liza Hadley, Nick Holliman, Kai Xu, Edyta Bogucka, Xinhuan Shu, Daniel Archambault, Kath Landgren, Ellen M. DeGennaro, Stephen M. Kissler

**Affiliations:** 1 University of Colorado Boulder, Boulder, CO, United States; 2 University of Cambridge, Cambridge, United Kingdom; 3 King's College London, London, United Kingdom; 4 University of Nottingham, Nottingham, United Kingdom; 5 Nokia Bell Labs, Cambridge, United Kingdom; 6 Newcastle University, Newcastle upon Tyne, United Kingdom; 7 Stanford University, Stanford, CA, United States

**Keywords:** communication, epidemiology, modelling, policy, visualization

## Abstract

Communicating scientific ideas to policy actors is a longstanding challenge, especially in epidemiological modeling where evidence is inherently uncertain. Central to this communication is visualization—the graphic representation of complex epidemiological modeling concepts through figures, plots, and charts. Effective model visualizations should be clear, simple, and easy to understand. This article brings theory from vision science to equip modelers in developing their understanding of and ability to assess and improve visualizations in their own settings. Designers can leverage fundamentals in vision science to aid their communication of modeling concepts to policymakers. We classify the different ways a modeling visual might fail and provide the necessary theory and examples for overcoming these problems in an epidemiological setting.

## 1 Introduction

Close communication between epidemiologists and policymakers is critical to successfully manage infectious disease outbreaks. Rarely do we formally consider our science communication practices (1). Visualizations (graphs, charts, figures, etc.) are central to this communication, providing a way to demonstrate different policy scenarios, clarify understanding, and find common ground. Over the past decades, and especially since the COVID-19 pandemic, a significant body of literature has been dedicated to the visualization of quantitative information (2) but little to the visualization of epidemiological modeling in particular. Epidemiological modeling uses mathematical, statistical, and/or computational tools to study how infectious diseases spread through different populations (3, 4). Epidemiological model visualization faces distinct requirements and constraints compared to general data visualization. In this article, we describe how epidemiological model visualization differs from other types of visualization tasks, examine what makes for strong policy-oriented epidemiological model visualization, and discuss how the effectiveness of model visualizations can be measured and assessed.

Previous literature on epidemiological model visualization has examined which aspects of figures and graphs policymakers reported to be most helpful, highlighting simple improvements modelers can make (5–8). Here, we aim to go one step deeper, digging into the theory of vision science and applying to epidemiological modeling. We aim to equip interested modelers with the theory and understanding for improving visualizations in their own settings.

## 2 The roles and requirements of epidemiological model visualizations

Epidemiological model visualizations serve a number of roles, such as reducing cognitive load (using visual perception instead of working memory), reducing dimensionality, crafting narratives, and adding context. These visualizations naturally need to capture uncertainties. Multiple sources of uncertainty occur in the modeling process and visualization can help summarize and highlight the different types of uncertainty to policy makers. Aleatoric uncertainty (9) (i.e., stochastic uncertainty, natural randomness) is irreducible, while epistemic uncertainty (10, 11) (missing knowledge, missing data) could be reduced with additional effort. Uncertainty is inherent in modeling, and the role of modeling process visualization is to clearly present options to policymakers so that they are conscious of the limits of the predictions (12, 13). It is important to consider which aspects of the uncertainty to highlight: for example, the naive approach of depicting a mean epidemic trajectory with credible bands may be less useful than highlighting low-probability but high-impact (i.e., worst-case) scenarios.

Epidemiological models, and thus epidemiological model visualizations, generally serve two main roles: forecasting, in which explicit quantitative predictions are made regarding the epidemic's near-term trajectory; and scenario modeling, where the aim is to understand longer-term consequences, qualitative impacts of potential courses of action, and the influence of underlying assumptions about the pathogen and its modes of transmission (14). Accomplishing these different objectives may require different visualization choices: for example, forecast visualizations may focus more on model outputs, treating the model itself as a “black box”, while scenario visualizations may treat the model as the central object of study, using visualizations to highlight the model's structure and how it impacts the projected epidemiological outcomes.

Beyond epidemiology, model visualizations arise in various fields, including clinical medicine, meteorology, and military science (15, 16). In meteorology for example, the US National Hurricane Center and other meteorological agencies produce maps with uncertainty cones and spaghetti plots capturing possible trajectories of storms, all based on underlying models (15). In military settings, 3D model visualizations of air defense systems help operations staff understand weak spots and potential threats (16). Despite the broad use and impact of model visualizations, there is little formal theory on the unique goals, methods, and evaluation strategies that pertain specifically to visualizing modeling, especially in the context of epidemiology.

One close analog is hurricane modeling, which shares constraints around speed of production (models may need to be generated, interpreted, and acted upon within days), ensemble approaches, and the communication of uncertainty (17–20). However, epidemiological model visualization has received comparatively less attention and has features that differentiate it somewhat from other types of model visualization: the diversity of underlying models (in contrast to e.g., meteorology, our field lacks canonical models), and the fact that the visualization itself can directly impact an epidemic (motivating future behavioral responses and hence transmission, whether unintended or not). The lack of canonical models for example can lead to less shared understanding that creates a burden of translating one model's implications into the language of another model, tradeoffs between tailoring visualization to the specific context and specific model vs. actively trying to create a research subfield culture where certain kinds of visualization are expected, and lastly can be perceived as less objective due to a lack of a default. For the behavioral feedback loop, tradeoffs between supporting a behavioral recommendation (e.g., lockdown, masking) and communicating complexity/uncertainty have higher stakes.

For visualizations, these features imply first a need to standardize what data needs to be communicated from models regardless of their type, from which we can develop visualization standards based on evidence of their effectiveness. Secondly, a need to develop visualization standards that are scientifically evaluated against expected communication tasks to hope to identify where miscommunication may occur in advance. Rather than expecting to produce one visualization recommendation for everything, we suggest developing a set of evidence-based designs and visualization evaluation tools. In this way, communications teams will have options to choose from, and tools to help test the basic operational effectiveness of those visualizations before they are published.

**Figure 1:**
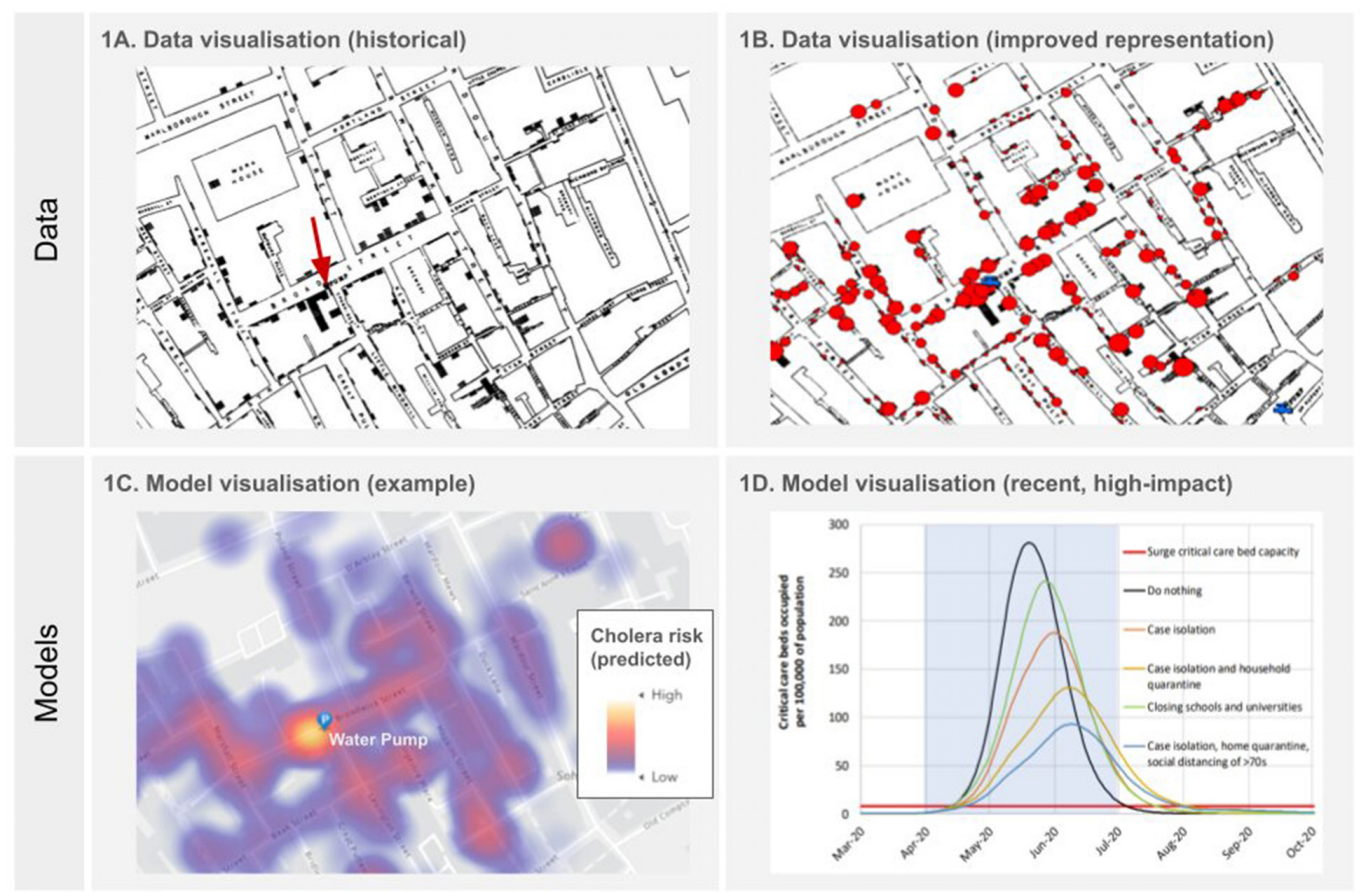
An example of the spectrum from data visualization to model visualization. **(A)** John Snow's 1855 map of the Broad Street cholera outbreak (22). This visualization demonstrates policy relevance with its then-novel geospatial layout of case counts (1 bar = 1 death). An alternative version is shown in **(B)**, reproduced with permission from Wilson (24). Here deaths are shown on a larger relative volume scale, and with the pump more clearly marked in a different color with its own symbol. The visualizations in **(A)** and **(B)** differ from what an example model visualization might look like **(C)**, if John Snow were working in present day and wished to inform current policy-makers about the risk of cholera to residents located at the addresses highlighted in the heatmap. Rather than empirical data, the model visualization in **(C)** instead needs to present projected risk estimates with uncertainty. The map in **(C)** was generated in ArcGIS, using Esri Tutorials, ^©^Esri, under CC BY-NC-SA license (25). **(D)** A more recent graph from Imperial College London's COVID-19 modeling team ‘Report 9' in 2020 that had an influential impact, likely due to its design simplicity and clear policy-relevance. Plot depicts mitigation strategy scenarios for Great Britain showing critical care (ICU) bed requirements. Reproduced from Ferguson et al. (23). Further use of visual dimensions (e.g., line thickness, shape, color contrast—see Figure 2) could have further increased its readability.

Model visualizations have played an important role in outbreak management and response. An example of the spectrum of influential data/ model visualizations is depicted in Figure 1. Some of the seminal texts in quantitative epidemiology contain model visualizations, like John Snow's 1,855 map of the central London Cholera outbreak (21, 22). In present day, early projections of the degree to which COVID-19 incidence could exhaust healthcare resources prompted lockdowns in the US and the UK (23). By concisely representing possible future scenarios, these epidemiological model visualizations helped establish a rapid understanding of the potential magnitude of each threat (Figure 1D).

Three main goals for epidemiological model visualization are apparent from prior work: (1) rapid interpretation for model users (5); (2) iterative model development (30); and (3) to provide model users with a way to communicate model insights to others (retranslation) (6). Drawing upon theory from vision science, cognitive science, and various applied fields, we examine:

(a) ways in which visual communication may break down between a modeler and policymaker (3.1),
(b) theory informing how epidemiological modeling can be best communicated visually to policymakers (3.2),
(c) interactivity in model-based science-policy communication (3.3),
(d) how to assess and improve a visualization (3.4),
(e) an example study design (3.5).

## 3 What makes for a good model visualization, and how do we know?

### 3.1 Communication breakdown

To begin understanding what makes for a good model visualization we can examine the opposite, i.e., what causes model visualizations to fail (Table 1). One major source of failure is visual design complexity: visualizations may be difficult to interpret due to overcrowding or poor use of color, spacing, patterns, or grouping. Insights from vision science (Section 3.2) and associated visualization tools (Section 3.4) have made significant headway toward overcoming these challenges, yet their implementation for epidemiological model visualization remains limited. Notably, there can be tradeoffs between minimalist and more embellished visual design approaches; for example, Borkin et al. found that visual embellishment can improve memorability, which may be particularly relevant where visualizations must communicate to multiple audiences simultaneously, including modeling experts, policymakers, and the general public (31).

**Table 1:** Types of communication breakdown to be mindful of in epidemiological model visualization.

Communication breakdown in epidemiological model visualization	
Type	Possible solutions and supporting literature
Design complexity (crowding, poor use of color etc.)	Extensive research on design principles in vision science. See section 3.2. Tools have also been developed to enable modelers to measure design complexity of their own figures, for example (26–28).
Misrepresentation	Visual understanding is discussed in for example (29). Providing additional model scenarios or interactive visualizations may also aid model users (Section 3.3).
Lack of policy-relevant metrics	Consider presenting results in terms of language and metrics that are familiar to the policy individual. See for example recommendations from policymakers for presenting epidemiological modeling (5–8).
Conflicts with pre-existing beliefs	Allow time for new concepts to be absorbed and for belief systems to be updated. Additional supporting model figures may help.

A second potential source of failure is misrepresentation - if the plot does not accurately represent the concepts and narrative it is trying to convey. In environmental systems modeling for example, this misrepresentation has been categorized as either ‘confusion regarding a concept' or ‘difficulty picturing the overall system' (32). In training epidemiological modelers, there is little emphasis on the visual representation of epidemiological concepts. Relatedly, even when policy actors may be comfortable with the concepts of the modeling visual, communication may fail. For example, outputs may not be presented in policy-relevant terms. Tension may also arise if model findings disagree with prior beliefs, motivating the need for additional supporting figures.

These four classes of visual communication breakdown are summarized in Table 1, with examples for overcoming each class.

### 3.2 Designing visualizations: the fundamentals

**Figure 2:**
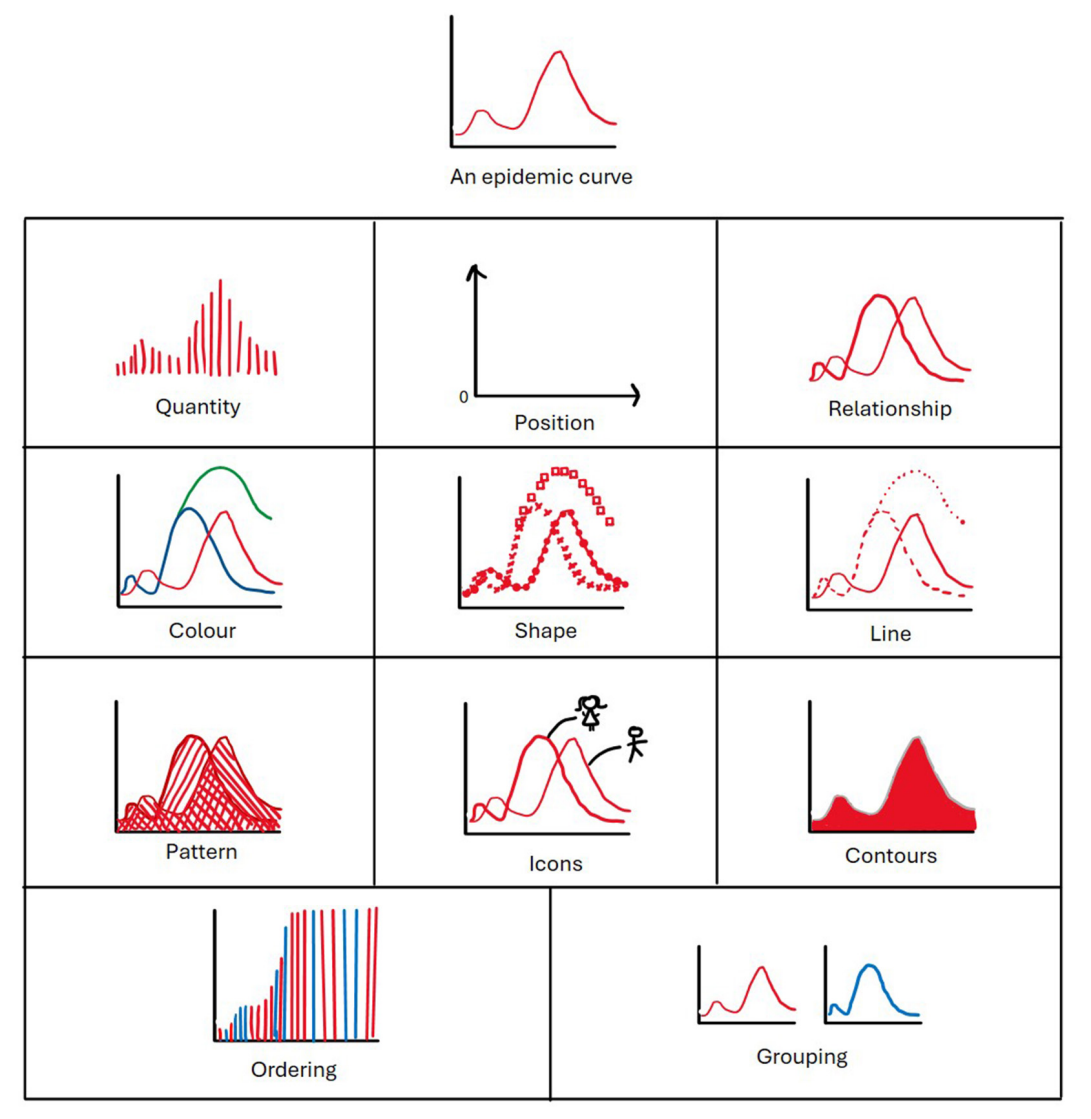
Components of a visualization, using an epidemic curve to illustrate. Diagrammatic dimensions include quantity, position, and relationship. Visual dimensions include color, shape, line, pattern, icons/isotypes, and contours. Structuring dimensions include ordering and grouping of information. Terminology follows that of Hil & Lachenmeier (34).

Drawing upon fundamentals in vision science, one should first identify three things: user, information, task (33). Here our user is a policy maker or decision maker, the information would be model outputs/processes (potentially from multiple models with different parameters) and any relevant contextual data, and example tasks could include exploring the impact of possible public health interventions or forecasting future cases. A new or adapted visualization will likely be needed any time the user, information, or task changes. In vision science, there is also a distinction between different dimensions or aspects of a graph, namely diagrammatical dimensions (quantity, position, relationship between variables), visual dimensions (color, shape, line, pattern, contours, isotypes/ icons), and structuring dimensions (ordering all outputs, grouping subsets of outputs) (Figure 2) (34). One can leverage these areas of visualization theory when communicating models for policy. For further ideas on faster or slower processing activities, color vs. shape, use of grouping, highlighting and annotation, and other Gestalt principles, we direct the interested reader to Franconeri et al. (35).

Alternatively, visualizations can be classified by level of user input: they can be static, dynamic, or interactive, to varying degrees. A static visualization might be included in a paper document. Interactive visualizations can range from the inclusion of hyperlinks, toggle switches to hide/show various data layers, to a full control-room set of inputs that the user can adjust and see the impact on model outcomes.

Other studies in epidemiological modeling have identified recommendations when creating visuals for policy actors, focusing on how information is reportedly received and understood by policy makers. They suggest ensuring visuals from modeling are as simple as can be to convey the required message, to use consistent style and format week-to-week, avoid overlaid charts in favor of separate plots, and that presenting model outputs as scenarios or options side-by-side were often found to be helpful (5–7). The recommendation of simple visuals in part stems from perceptual and cognitive limits on memory and information flows. The recommendation of consistency can be viewed as a form of training (training the policymaker on the visual). Training and experience significantly influence perception and cognition outcomes (36). There is value in substantiating these suggestions further to fill current knowledge gaps.

### 3.3 Interactivity

Traditional science-policy communication follows the central idea of the scientist presenting a coherent narrative from model outputs and optionally receiving verbal or written feedback, follow-up of questions, or other responses. Model outputs are generally gatewayed by the scientists. Consider here an alternative: where policy actors can, if they so wish, engage directly with the visualization through interactive components such as sliders, toggles, dropdown filters, and parameter controls. This may be appropriate for specialist public health policymakers looking to gain deeper epidemiological understanding (compared to more high-level decision makers who have a broader remit). Interactivity in this way is distinct from principles of static design which have dominated the model visualization literature (37).

Some interactive tools for epidemiological models do exist [for example (38–40)], although the primary mechanism for communication is still static plots and figures. Interactive visualizations, used thoughtfully, open the possibility to qualitatively change the role that the model plays in mediating a scientist-policymaker conversation. Interactivity permits the interested policymaker and modeler to query the model at the same time, parallelising inquiry, and possibly making the process of developing new insights and scenarios more rapid. The visualization could serve as the scaffold for the conversation, and the modeler (or other knowledge broker) can help interpret and contextualize model results. By observing how the policymaker interacts with the model visualization, the modeler may also identify ways of improving it, accounting for scenarios that might not have been previously considered. Modeler and policymaker could both have agency on insights.

Moreover, interactivity can open avenues for different, and arguably deeper, types of understanding (41). Philosopher Gilbert Ryle introduced the distinction between ‘knowing that' and ‘knowing how' (42). ‘Knowing that' is a type of intellectual knowledge from reading books, listening to reports, and passively observing the world. In the epidemiological context, one might for example know that relaxing restrictions on gathering sizes could increase the growth rate of an epidemic by 1.4 times. ‘Knowing how' in contrast refers to an ability to link cause and effect, without necessarily knowing the mechanisms by which the two are connected. It is associated with a type of embodied knowledge, intuition, or phronesis, depending on context. Knowing how is developed by interacting with the world, actively embodying propositional questions (if I do *x*, what will happen?) and hypotheses. Both types of knowledge are important, yet ‘knowing how' is arguably more important for those who seek future impact based on present knowledge. It also shortens the iteration time for propositional investigation (rapid hypothesis testing)—how does a person get a ‘feel' for something when they don't understand how it works mechanistically?

The central importance of both ‘knowing that' and ‘knowing how' is embodied in the training programs of many professionals. Medical students begin with pre-clinical book training—establishing a basis of ‘knowing that'. They then engage in actual practice, e.g., through observed structured clinical exams (OSCEs), where the patient-actor serves as an interactive ‘model' with which the student engages. Equivalently, pilots learn about the inner workings of an airplane, then develop a know-how for flying it using simulators—another type of interactive model. A similar aim seems worthwhile to pursue for specialist policymakers seeking to deepen their understanding of infectious disease outbreak control. Epidemiologists can serve a critical role in helping the policymaker ‘know that' epidemics behave in certain ways. Interactive models may open the possibility of layering ‘know-how', allowing the interested policymaker to tinker with a model visualization, observe its behavior, and develop an intuition for how certain actions might lead to different future scenarios.

Many pitfalls face interactive model visualizations however and these must be carefully weighed before embarking on the significant effort it takes to create such things. Interactivity can be at odds with simplicity, potentially muddying the visual explanation. It can also be challenging to craft a single, concise visual narrative, since the user is free to explore the visualization in any order and in unanticipated ways. This could hinder understanding and make it more difficult to achieve consensus. It can also be more challenging to assess the user experience and impact of interactive visualizations due to their complexity. Thus, interactive visualizations should be seen as a potentially valuable option, but not necessarily as an ideal for model visualization.

### 3.4 Measuring and assessing effective model visuals

A timely question in epidemiological response work is ‘how do you evaluate a public health intervention?'. Evaluation enables identification of progress, pitfalls, and gaps for the future. Equivalently, for communicating modeling, we ask ‘how do you evaluate a model visualization?'. How can we measure model visualizations that are effective and well-understood by policy actors? This can be done quantitatively by measuring for example user response time or ‘learning time' and retrieval/ recall accuracy. Other visual design metrics can also be informative, such as contrast sensitivity and visual saliency (is the eye drawn to the correct parts of the graph, do key scientific messages stand out?), edge congestion (is there clear spacing?), and color analysis (does the visual use colors clearly?) (43). Tools are being developed that enable modelers and other designers to easily test their graphics against these visual design ‘quality metrics' (VizQM) (26–28).

Graphs and plots can also be assessed qualitatively, for example, through user opinion and measures of understanding. User opinion could include surveying ‘which graph did you find clearest/ easiest to understand?' or ‘which graph did you prefer and why?', and deeper questions on utility: ‘to what extent does the model visualization answer the questions it is confronted with?'. Note that these policy questions may not be known explicitly in advance. The modeling may have different goals depending on the timing of an epidemiological event such as showcasing qualitative differences in scenarios vs. exactly predicting ICU beds.

For measuring the complexity of a graphic, we may also draw insights from linguistics. Looking to analysis of policy briefs and other written documentation, a recent study partitions linguistic complexity into two: semantic complexity (‘high average word length or sentence length') and conceptual complexity (‘difficulty in comprehending a message due to fundamentally not understanding certain words or how things are related, for example difficult ideas, jargon, things you've never heard') (44). Can we break down visual complexity in this way? An equivalent could be visual design complexity (the traditional measures of crowding, color analysis, etc. as above) and conceptual complexity (unfamiliar or uncommon diagrammatic dimensions, graph formats with an assumed knowledge for interpreting such as log scales, etc.). This could be another way to assess the effectiveness of visuals.

In Table 2, we summarize the above metrics for assessing effective model visualizations by grouping into those that measure the visual, those that measure the user, and those that measure subsequent real-world change or action.

**Table 2:** Example metrics for assessing effective visualizations. Note that while measuring subsequent real-world change is a valid assessment criteria, for science informing policy, this is often not a direct goal - the role of science is usually to inform not persuade.

Measuring the visual	Measuring the user	Measuring real-world change
Contrast sensitivity	User response time/learning time	Adoption rate (e.g., becoming the go-to visualization format)
Edge congestion	Retrieval/recall accuracy	
Visual saliency	User opinion^*^	
Color analysis	Demonstrated/written understanding	
Conceptual complexity		

^*^User opinion should be treated carefully—the role of individual differences makes user testing difficult and often unrepeatable.

### 3.5 Designing studies to assess epidemiological model visualizations

Previous studies have utilized qualitative interviewing and stakeholder surveys (5–8, 45). Experimental design studies enable deeper learning. In the context of model visualization, the speed and depth of understanding play an important role. A hypothetical study design is as follows:

Recruit relevant public health policy and decision makers, i.e., those who either already use epidemiological modeling in their routine or outbreak response work or are interested to begin using modeling. Participants are shown a series of static epidemiological plots, scenarios, or forecasts in turn. Pairs of plots could be shown side-by-side varying a key visual dimension each time. For each of the plots, the participant could be asked to rate the degree to which they feel they grasp the underlying meaning of each visual, which they find easiest to understand, and/or to produce a text answer of learnings from the plot as if they were writing a policy brief for a colleague. These indicators can be assessed for user preference, or from the text response, accuracy and completeness of understanding and ability to retranslate key results to other policy actors. Analysis of all responses could inform diagrammatic dimension choices (spacing, color, grouping, etc.) for creating and explaining epidemiological model insights in the future. A study of this style could also be extended to include text analysis such as figure titles or legends accompanying the modeling visuals.

An equivalent study could also be carried out comparing static, interactive, and dynamic (i.e., video) visualizations of the same epidemiological concept. Analysis of responses would inform possible strengths and weaknesses of engaging policy actors in direct interactive model visualization, as described in section 3.3.

More broadly, these examples motivate key considerations for designing studies on the effectiveness of model visualization: effectiveness of a visualization based on user preference/ adoption, effectiveness as efficiency, effectiveness as understanding, effectiveness as trust, and (if meaningful and measurable) effectiveness on downstream policy recommendations.

## 4 Looking forward: advice for modelers

This commentary has introduced a range of visualization concepts for use in epidemiological modeling and policy. Epidemiological modelers wishing to apply this theory to their own work are advised of the following:

1. Ensure figures have simple visual design, faithfully represent their main concept, and include policy-relevant metrics. Fundamental design principles are described in the main text.
2. For more depth and theory, engage with the vision science literature cited throughout this article.
3. Experiment with interactivity, but be wary of information overload.
4. Consult with or seek out collaborations with visualization experts. Interested readers are directed to The Alan Turing Institute and other initiatives (46).
5. If interested, pursue formal research on visualization effectiveness in the context of epidemiological modeling for policy. There is a need to regularly research and assess these aspects of science communication and modeling-policy practices.

## Data Availability

The original contributions presented in the study are included in the article/supplementary material, further inquiries can be directed to the corresponding author.
